# Effects of Climate Change on Habitat Availability and Configuration for an Endemic Coastal Alpine Bird

**DOI:** 10.1371/journal.pone.0142110

**Published:** 2015-11-03

**Authors:** Michelle M. Jackson, Sarah E. Gergel, Kathy Martin

**Affiliations:** 1 Department of Forest and Conservation Sciences, University of British Columbia, 2424 Main Mall, Vancouver, British Columbia, V6T 1Z4, Canada; 2 Environment Canada, 5421 Robertson Road, Delta, British Columbia, V4K 3N2, Canada; Clemson University, UNITED STATES

## Abstract

North America’s coastal mountains are particularly vulnerable to climate change, yet harbour a number of endemic species. With little room “at the top” to track shifting climate envelopes, alpine species may be especially negatively affected by climate-induced habitat fragmentation. We ask how climate change will affect the total amount, mean patch size, and number of patches of suitable habitat for Vancouver Island White-tailed Ptarmigan (*Lagopus leucura saxatilis*; VIWTP), a threatened, endemic alpine bird. Using a Random Forest model and a unique dataset consisting of citizen science observations combined with field surveys, we predict the distribution and configuration of potential suitable summer habitat for VIWTP under baseline and future (2020s, 2050s, and 2080s) climates using three general circulation models and two greenhouse gas scenarios. VIWTP summer habitat is predicted to decline by an average of 25%, 44%, and 56% by the 2020s, 2050s, and 2080s, respectively, under the low greenhouse gas scenario and 27%, 59%, and 74% under the high scenario. Habitat patches are predicted to become fragmented, with a 52–79% reduction in mean patch size. The average elevation of suitable habitat patches is expected to increase, reflecting a loss of patches at lower elevations. Thus ptarmigan are in danger of being “squeezed off the mountain”, as their remaining suitable habitat will be increasingly confined to mountaintops in the center of the island. The extent to which ptarmigan will be able to persist in increasingly fragmented habitat is unclear. Much will depend on their ability to move throughout a more heterogeneous landscape, utilize smaller breeding areas, and survive increasingly variable climate extremes. Our results emphasize the importance of continued monitoring and protection for high elevation specialist species, and suggest that White-tailed Ptarmigan should be considered an indicator species for alpine ecosystems in the face of climate change.

## Introduction

Climate-related range shifts are discernable across the globe for many animal species [1] and clear fingerprints related to climate change are detectable on bird species assemblages [2,3]. Mountain ecosystems are among the most at risk from climate change [4]. Mountains often have high levels of biodiversity and harbour large numbers of endemic species [5,6], many of which are restricted to alpine habitats [7]. Such habitats are gradually being lost due to tree and shrub encroachment [8,9], resulting in range contractions of alpine species at their lower altitudinal limits [10,11]. With no room “at the top” for upward expansion, alpine species face habitat loss and fragmentation and, ultimately, risk of extinction [12–14]. Increased competition from invasive species and low elevation generalists may further accelerate population declines for alpine specialist species under a warming climate [15,16].

Coastal temperate mountains are particularly vulnerable to climate change as they occur at lower elevations compared to interior mountains and contain a smaller area of alpine habitat [17]. Relatively little is known about the importance of alpine ecosystems for avian biodiversity, and most large-scale bird sampling schemes, including citizen science programs such as eBird and the Breeding Bird Survey, lack adequate reports from high elevations because of their relative inaccessibility and low population densities [18,19]. However, British Columbia’s coastal mountains, including those on Vancouver Island, harbour a remarkable diversity of birds, many of which are of conservation concern and threatened by climate change ([20], Boyle and Martin, *In Review*). Other threatened wildlife taxa depending on these coastal montane ecosystems are similarly difficult to study, such as mountain goats (*Oreamnos americanus)* and the critically endangered, endemic Vancouver Island Marmot (*Marmota vancouverensis*; [21]). In general, North America’s coastal alpine are remarkably understudied, in part due to their extremely rugged terrain and restricted road and trail access.

Over the past decade, the Pacific Northwest of North America has experienced a significant warming trend (roughly 0.5–1.5°C; [22]). Temperatures are predicted to continue to warm across all seasons at rates comparable to or higher than current rates, with variable predictions for precipitation [23]. Such changes will accelerate woody vegetation growth in former alpine tundra; for instance, Flower *et al*. [24] predict rapid upward shifts in elevational range for both Douglas Fir (*Pseudotsuga menziesii*) and spruce (*Picea* spp.) in British Columbia, and Laroque *et al*. [25] used dendroclimatological analysis to document climate-related pulses of tree establishment at high elevations on southern Vancouver Island during the 20^th^ century. They predicted rapid infilling of alpine meadow sites within 10–20 years, with negative consequences for montane meadow-dependent wildlife. Subsequent research has shown that infilling by trees and shrubs at high elevations occurred on Vancouver Island between 1962 and 2005 [26]. Such expansion of woody vegetation leads not only to loss of suitable habitat for alpine species but also to habitat fragmentation and loss of connectivity. Most studies investigating potential climate-induced range changes for wildlife focus primarily on habitat loss and shifting climate envelopes, but fragmentation may also play a crucial role, particularly for alpine species with limited room “at the top” for tracking climate envelopes upward in elevation.

The Vancouver Island White-tailed Ptarmigan (*Lagopus leucura saxatilis*; hereafter VIWTP) is endemic to Vancouver Island and was designated a subspecies in 1939 based on unique morphological characteristics [27,28]. An alpine specialist bird that spends its entire life history above the treeline, VIWTP was blue-listed as vulnerable by the British Columbia government in 1992 given its endemic status and low density [29]. White-tailed Ptarmigan are adapted to cold environments and cannot physiologically tolerate hot temperatures [30,31]. They rely on permanent snow fields for thermoregulation [31,32] and areas with sufficient moisture (from summer precipitation and snowmelt) for food such as ericaceous shrubs, grasses, and forbs [33]. Because the coastal alpine comprises lower elevations than interior mountains, VIWTP habitat exists as smaller, more fragmented “sky island” patches than interior White-tailed Ptarmigan habitat on the mainland. For example, the size of alpine patches comprehensively surveyed for VIWTP in 1997 varied from 0.14 to 7.10 km^2^ (KM unpublished info) versus 2.7 to > 13.2 km^2^ in Colorado [34]. Although ptarmigan have persisted on Vancouver Island, there is likely a demographic cost to utilizing smaller habitat patches. For instance, VIWTP in the central island (with larger, more continuous patches of alpine) had higher breeding success and higher adult survival than birds in the more fragmented south island populations in 2011 [33].

Because of their physiological requirements and the fact that their habitat exists as smaller alpine patches at lower elevations than mainland White-tailed Ptarmigan [35,36], VIWTP are threatened primarily by climate change. Wang et al. [37] modeled the relationships between 44 climate variables and the distribution of 16 biogeoclimatic zones in British Columbia. They found that the Coastal Mountain-heather Alpine zone, upon which VIWTP primarily depend, is predicted to decline by nearly 50% by the 2080s based on a single consensus map that incorporated 20 climate change scenarios, with near total loss predicted for Vancouver Island. They predict alpine ecosystems will be replaced by Coastal Western Hemlock forests, potentially resulting in increased forest productivity and carbon sequestration at the expense of habitat for alpine specialist species [37]. Survey efforts combined with museum records and other historic sources revealed that VIWTP still occupied the same mountains on Vancouver Island in 2004 as they had occupied historically (early to mid-1900s; [35]). However, this study was coarse in scale and did not examine the effects of climate or topography on ptarmigan habitat. As climate change accelerates, adequate conservation of VIWTP will ultimately depend on detailed knowledge of the amount and configuration of suitable breeding habitat predicted under current and future climates.

Species distribution models can be a useful tool for assessing the impacts of environmental change and the distribution of organisms. In a previous study describing the quantity and location of suitable ptarmigan breeding habitat on Vancouver Island, we showed that species distribution models trained using opportunistic observations submitted by hikers (citizen science) are comparable in quality and predictions to models trained using time- and cost-intensive field survey data [36]. Thus, although large-scale avian citizen science platforms such as eBird may not adequately capture observations from alpine regions, localized and species-specific citizen science programs can provide valuable information about elusive alpine species such as VIWTP. Here, we build on our comparison of five modeling techniques using citizen science and field survey input data to investigate how climate change will alter the size, distribution, and configuration of suitable VIWTP breeding habitat. Specifically, we ask how the total amount, mean patch size and number of patches of suitable VIWTP habitat will change by the 2020s, 2050s, and 2080s due to climate change. Our study identifies areas of highest vulnerability and guides future plans for conservation of this threatened subspecies, along with providing a baseline for future studies of connectivity for mountain wildlife across increasingly fragmented habitat.

## Materials and Methods

The ptarmigan banding work was approved by the UBC Animal Care Committee Protocol A01-0097. Birds were captured under the Canadian Federal Banding Sub Permit—10365BO and BC Provincial banding permit (Water, Lands and Parks)—C068541. Research was conducted in Strathcona Provincial Park under Park Use Permit—#3081065.

### Study area and species occurrence data

The study area encompassed all of Vancouver Island, located on the southwest coast of British Columbia, Canada (between 47° and 52° N latitude and 123° and 128° W longitude). At 460 km long (north to south) and 80 km wide (east to west; 32,134 km^2^), Vancouver Island is the largest island on the west coast of North America. Elevation varies from 0 to 2,195 m a.s.l. A central spine of mountains spans the length of the island, with highest elevations located in the center of the island within Strathcona Provincial Park (with four mountain summits between 1,830 m and 2,195 m). These mountains create a rain shadow on the eastern side, resulting in a strong east-west precipitation gradient across the island. The biogeography of the island has been classified into biogeoclimatic (BEC) zones by the provincial government that represent vegetation, soil, and climate conditions. Four BEC zones exist on Vancouver Island, including Coastal Douglas Fir, Coastal Western Hemlock, Mountain Hemlock and Coastal Mountain-heather Alpine. The largest areas of continuous alpine habitats are found in the central region of the island, while alpine patches in the northern and southern regions are generally lower in elevation and more fragmented.

We used two sources of distributional data for Vancouver Island White-tailed Ptarmigan. The first consisted of ptarmigan presence locations from field surveys conducted by K. Martin from 1995–1999. These surveys targeted the entire range of VIWTP and representative alpine habitat deemed suitable for ptarmigan was searched in the north, central, and south island. Birds were located using playbacks of male territorial calls and chick distress calls, then captured with a snare attached to an extendable pole [38] and outfitted with a necklace collar radio transmitter (RI-2D/2B, 18 mo battery life, weight 6–9 gm, Holohil Systems Ltd., Carp, Ontario). K. Martin and field crews tracked the radio-collared birds over the 4-year period by foot during summer and recorded encounters with other uncollared birds using playbacks and incidental encounters. GPS coordinates were recorded at each bird location.

From this field survey dataset, we extracted summer (June–October) observations and eliminated sightings of juveniles, since they were usually found at or near the same GPS coordinate as their parent during the summer period. We then eliminated duplicates at the same GPS point (i.e., if a flock of several birds was recorded at one point, only one observation was included in the final dataset). To minimize bias due to certain individuals being observed more than others, we first eliminated records from the same individual that were < 1 day apart, and then randomly selected two records per individual. Lastly, we randomly selected one observation within each 100-m grid cell of the environmental predictor GIS layers. All filtering of the original ptarmigan data was carried out using R 3.1.2.

**Fig 1:**
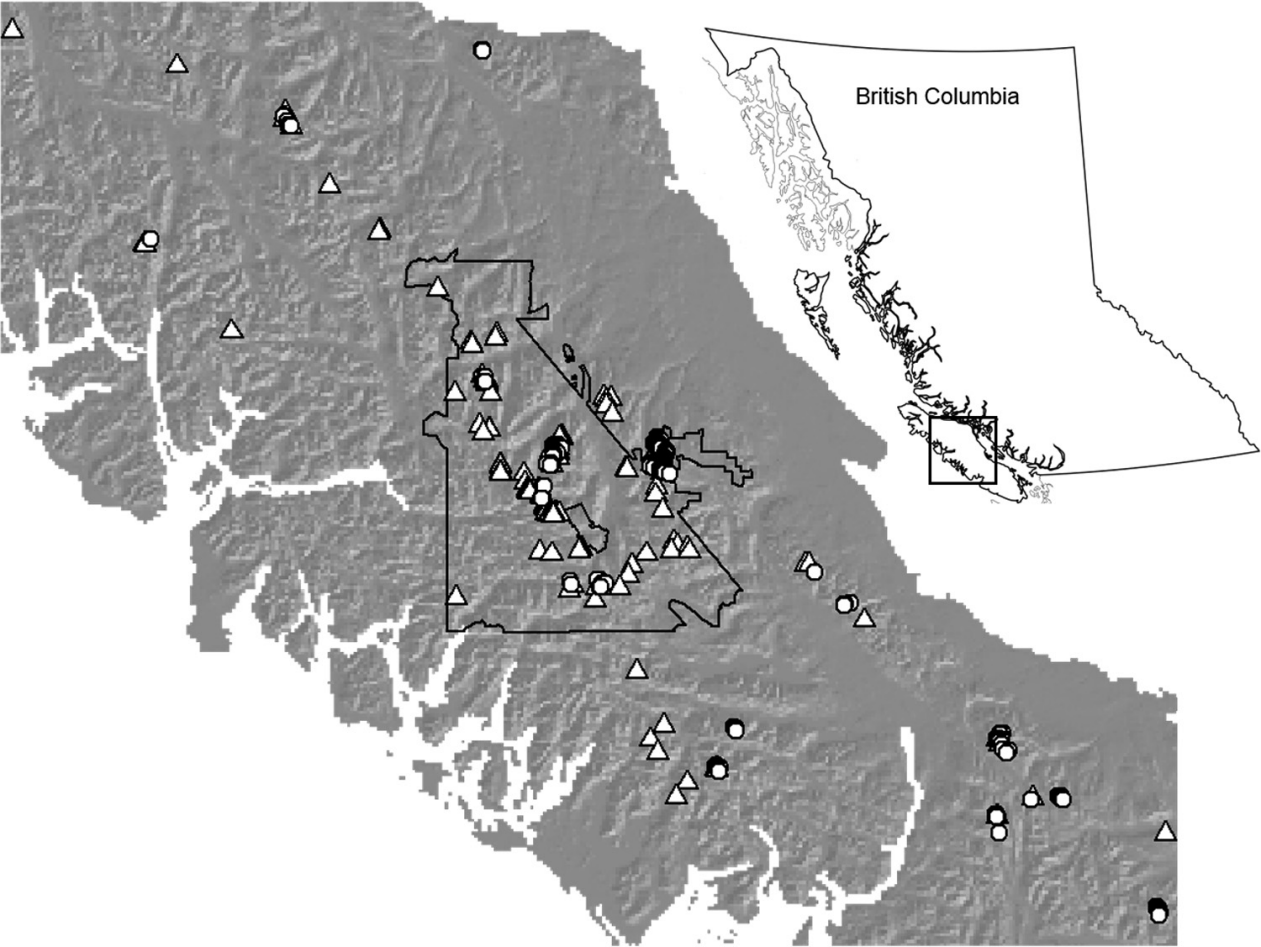
White-tailed Ptarmigan presence locations on Vancouver Island from the field survey (circles; N = 207) and public submissions (triangles; N = 137). Strathcona Provincial Park is outlined in black. This map is similar but not identical to that shown in [36].

In order to supplement the field survey records, an opportunistic citizen science program was initiated in partnership between the Strathcona Wilderness Institute and K. Martin at the Centre for Alpine Studies, University of British Columbia. Notices were posted at trailheads within Strathcona Provincial Park describing distinguishing features of White-tailed Ptarmigan. The notices requested that hikers report their ptarmigan sightings by mailing a card or sending an e-mail with GPS or map coordinates and photos (if possible) to K. Martin at UBC. The citizen science initiative began in 1995 and continues to the present, currently resulting in 404 confirmed White-tailed Ptarmigan sightings (see [36] for details). Although trailhead cards were posted within Strathcona Provincial Park, many hikers frequently submitted records from their other alpine hikes on the island after finding out about the program. This resulted in a public dataset spanning the entire range of alpine habitat on Vancouver Island (Fig 1).

We extracted summer (June–October) observations from the public dataset and then filtered the observations by location accuracy, keeping only those that contained precise GPS or map coordinates (accurate to within 100 m). As with the field survey observations, we eliminated duplicates at the same location and randomly selected one observation within each 100-m grid cell. Because the field survey and public datasets result in models with similar accuracy and distributional predictions when using machine learning modeling techniques [36], we combined ptarmigan presence locations from each dataset in order to maximize sample size and model accuracy for the purpose of this study. If two sightings fell within the same 100-m grid cell in the combined dataset, we randomly eliminated one sighting from the cell. The resulting combined dataset contained 344 sightings (207 from the field survey and 137 from the public dataset; Fig 1).

Because we lack information on ptarmigan absences (areas traversed by observers where ptarmigan were not encountered) in either dataset, we generated a baseline random sample of pseudo-absences for the combined dataset to represent the landscape potentially available to VIWTP. First, we constrained the pseudo-absence background within a 200-m buffer surrounding the minimum convex polygon around the entire set of ptarmigan observation points. Within this area, we restricted the background to areas within the Coastal Mountain-heather Alpine and Mountain Hemlock Biogeoclimatic (BEC) zones. The majority (92%) of ptarmigan presence locations fell within the Coastal Mountain-heather Alpine BEC zone, while the remainder fell within the Mountain Hemlock zone (except for one sighting in the Coastal Western Hemlock zone).

Some methods for generating pseudo-absences first predict low suitability areas using presence-only models and select pseudo-absences from those areas [39–41]. Senay et al. [42] apply a novel three-step approach to select pseudo-absences using environmental constraints which can outperform models where pseudo-absences are randomly generated. We used distribution maps of BEC zones, which incorporate environmental variables known to be correlated with ptarmigan presence, to define environmental constraints for pseudo-absence selection. In contrast to methods that generate pseudo-absences outside of suitable habitat, we differentiated ‘‘suitable” from ‘‘highly suitable” habitat by generating pseudo-absences within potentially suitable habitat (which included both Mountain Hemlock and Coastal Mountain-heather Alpine zones). Such a distinction is important for rare and threatened species [43]. Including Mountain Hemlock zones in our pseudo-absence background also allowed for better discrimination between presence and absence locations.

Within this background, we excluded all areas within a 200-m buffer around known ptarmigan presence locations (the median distance moved by ptarmigan within a day during summer was 170 m based on the radio-telemetry study; N = 37 movements). Finally, we randomly selected twice as many pseudo-absence locations as the number of ptarmigan presence locations (688 total pseudo-absences). This choice was based on an initial exploration of different ratios of presences to pseudo-absences (1:2, 1:4, and 1:10) to check for changes in model accuracy measures (sensitivity, specificity, Kappa, AUC, and the True Skill Statistic). Using a ratio of 1:2 resulted in the highest Kappa value, whereas all other accuracy measures were not affected by presence-to-pseudo absence ratio.

### Abiotic predictor variables

To model ptarmigan occurrence, we used eight abiotic variables believed to influence ptarmigan distribution (Table 1). Five topographic variables included elevation, aspect, slope, compound topographic index (CTI, commonly referred to as a wetness index), and a vector ruggedness measure (VRM). All topographic variables were derived from a 100-m resolution digital elevation model (DEM). Aspect and slope were calculated using ArcGIS Spatial Analyst. We transformed aspect as x = -1*cos[Ø(π/180)], where Ø is the aspect measured in degrees. Values ranged from -1 where the angle of solar incidence was lower (north-facing slopes) to 1 where it was higher (south-facing slopes). The importance of aspect depends on slope, so we assigned aspects with slopes of <5° a neutral value of 0 [44]. CTI was calculated based on the slope and upstream contributing area orthogonal to the flow direction [45], so that low values represent small catchments such as steep slopes or ridges and high values represent large catchments such as gentle slopes or depressions. VRM is an integrative measure of terrain heterogeneity based on slope and aspect, and was calculated using neighbourhoods of nine pixel cells. Ruggedness values can range from 0 (no terrain variation, or a smooth surface) to 1 (extremely rugged terrain with high variability in slope and aspect; [46]).

**Table 1:** Abiotic variables used in the Random Forest model for predicting the distribution of Vancouver Island White-tailed Ptarmigan habitat. The mean and standard deviation (SD) along with the range (in brackets) of each variable are shown for ptarmigan presences and pseudo-absences.

Variable	Description	Mean (± SD) and range of ptarmigan locations		
		Field survey presences	Public presences	Pseudo-absences
Elevation (m)	Elevation a.s.l.	1,557 (±178) [1,088–1,989]	1,590 (±189) [968–2,29]	1,173 (± 197) [675–1,800]
Aspect	Aspect transformed for solar incidence^1^	-0.50 (± 0.52) [-1–0.99]	-0.28 (± 0.60) [–1–1]	-0.08 (± 0.68) [–1–1]
Slope (°)	Slope of the landscape	21.3 (± 9.8) [2.1–48.4]	17.6 (± 9.8) [1.3–53.4]	22.1 (± 11.6) [0–57.2]
CTI	Compound Topographic Index^2^, a function of the slope and the upstream contributing area [45]	11.0 (± 0.9) [9.2–14.5]	10.9 (± 0.9) [8.9–14.3]	11.4 (±1.4) [8.9–19.4]
VRM	Vector Ruggedness Measure, a measurement of terrain ruggedness as the variation in three-dimensional orientation of grid cells within a 9-cell neighborhood [46]	0.063 (± 0.031) [0.006–0.162]	0.079 (± 0.035) [0.006–0.212]	0.049 (± 0.031) [0.001–0.218]
Mean summer precipitation (mm)	Mean annual summer (May–Sept.) precipitation	619 (± 390) [284–1,867]	594 (± 263) [152–1,591]	563 (± 300) [171–2,536]
Mean summer temperature (°C)	Mean summer temperature	10.3 (± 1.0) [7.7–12.8]	10.2 (± 1.1) [7.3–13.3]	12.0 (± 1.0) [8.8–14.5]
Precipitation as snow (mm)	Precipitation as snow from August in previous year to July of current year	1,793 (± 721) [586–4,592]	1,436 (± 522) [676–3,718]	1,101 (± 507) [330–4,129]

^1^ x = -1*cos[Ø(π/180)], where Ø is the aspect measured in degrees. Since the importance of aspect depends on slope, we assigned aspects with slopes of <5° a neutral value of 0 [44].

^2^ CTI = ln(As / tan(β)) where As = Area Value calculated as (flow accumulation + 1) * (pixel area m^2^) and β is the slope expressed in radians.

We extracted three climate variables for each ptarmigan location from the year the observation was collected using the program ClimateBC v5.03 [47] (http://cfcg.forestry.ubc.ca/projects/climate-data/climatebcwna/#ClimateBC; Table 1). ClimateBC extracts PRISM [48] monthly climate normal data at a coarse scale (800 x 800 m) and then downscales it using bilinear interpolation and adjustments based on latitude, longitude, and elevation. We used mean summer temperature, mean summer precipitation, and precipitation as snow to model ptarmigan distribution. These climate variables reflected prior knowledge about White-tailed Ptarmigan energy and water constraints [30–32]. To predict suitable ptarmigan habitat across Vancouver Island, we also extracted the climate variables for the baseline time period 1980–2010 for each cell in our 100-m DEM.

**Fig 2:**
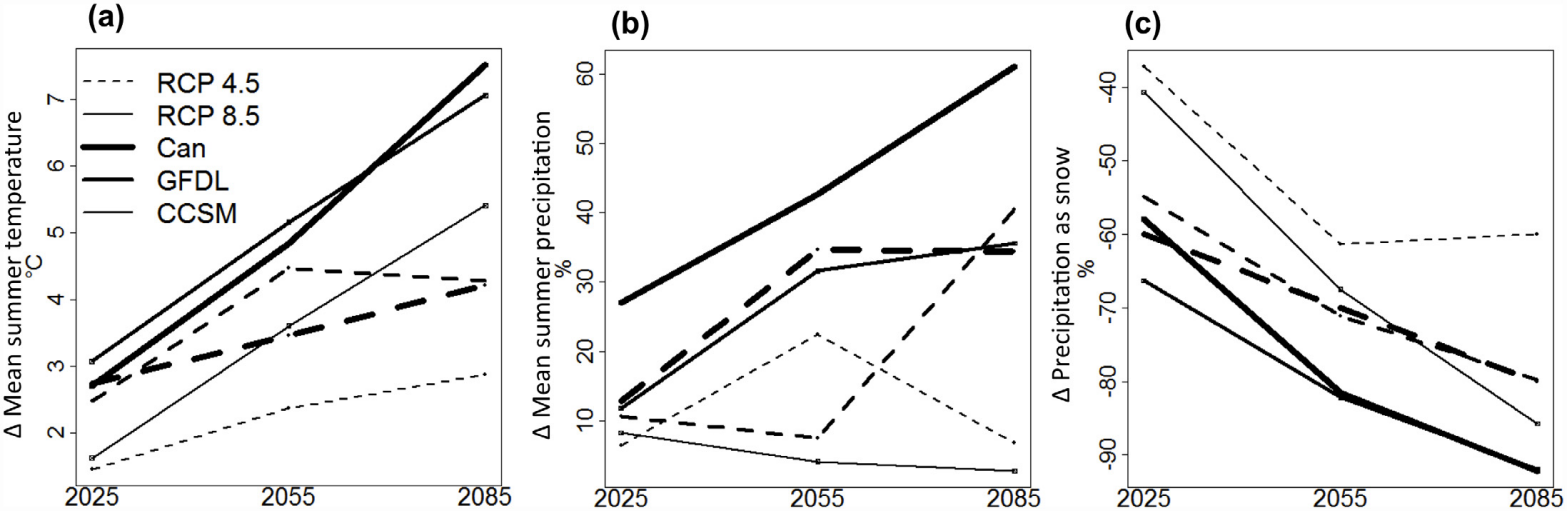
Predicted changes in mean summer temperature (a), mean summer precipitation (b) and precipitation as snow (c) according to three general circulation models (GCMs): CanESM2 (Can), CCSM4 (CCSM), and GFDL-CM3 (GFDL), and two IPCC emissions scenarios (RCP 4.5 and RCP 8.5) for Vancouver Island. The RCP 4.5 scenario is displayed using dotted lines while the RCP 8.5 scenario is displayed using solid lines. Line weights correspond to the three GCMs (thick = Can, medium = GFDL, thin = CCSM).

To model the distribution of VIWTP in the future, we extracted the above climate predictors from ClimateBC for the 2020s (2010–2039), 2050s (2040–2069), and 2080s (2070–2099) across three general circulation models (GCMs): CanESM2 from the Canadian Centre for Climate Modelling and Analysis, CCSM4 from the National Center for Atmospheric Research, and GFDL-CM3 from the Geophysical Fluid Dynamics Laboratory, and two Representative Concentration Pathways (RCPs) from the International Panel on Climate Change AR5 report: RCP 4.5 and RCP 8.5. We chose these three GCMs to represent a range of possible climate futures on Vancouver Island based on scatter plots of future temperature and precipitation using all 16 GCMs made available through ClimateBC. The GFDL model predicts a hot, moderately wet future, the CanESM2 model predicts a hot, very wet future, and the CCSM model predicts the least change in both temperature and precipitation (Fig 2). The RCP 4.5 scenario represents lower future greenhouse gas concentrations and more conservative predictions of climate change, whereas the RCP 8.5 scenario represents higher future greenhouse gas concentrations and a more extreme climate change scenario. In order to predict future ptarmigan distribution, we extracted the three climate predictors for every cell in the 100-m DEM covering all of Vancouver Island for each GCM and greenhouse gas scenario combination.

### Distribution model construction

We predicted probability of White-tailed Ptarmigan occurrence over all of Vancouver Island at 100-m resolution using a Random Forest model [49,50] with the ‘randomForest’ package in R. Random Forest models had high accuracy and were most consistent when modeling the field survey and public data separately [36]. Furthermore, Random Forest is robust to the inclusion of correlated variables (e.g., temperature and elevation; [49,51]). Random Forest models produce many classification trees, each constructed using a bootstrap sample of the input data. The number of predictor variables randomly selected at each tree node was set to the default (the square root of the total number of predictors), and 1500 classification trees were constructed.

We used ptarmigan presence and pseudo-absence locations from the pooled datasets as the response variable and all abiotic predictor variables in the model. In order to convert probability of presence into binary presence-absence predictions, we used the threshold probability that maximized sensitivity (percent of presences correctly classified) at 90% using the ‘PresenceAbsence’ package in R. Map pixels with probabilities above the threshold were considered ‘suitable’ habitat and those below the threshold were considered ‘unsuitable.’ We felt that from a conservation perspective, errors of omission (misclassifying presences as absences) were more “dangerous” than errors of commission (misclassifying absences as presences). Furthermore, since we knew the presences to be true presences but absences were pseudo-absences and may have been false (located in areas where ptarmigan are present) we chose to maximize the number of presences correctly predicted at 90%, recognizing that this may result in slight over-prediction of suitable habitat compared to other threshold-setting measures (e.g., maximizing kappa). We evaluated the RF model by calculating out-of-bag (OOB) errors based on bootstrapped samples of the input data according to the pre-chosen threshold using the R package ‘PresenceAbsence’. We constructed all models using R 3.1.2.

We predicted the RF model onto the entire landscape of Vancouver Island at 100-m resolution using baseline (1980–2010) and future (2020s, 2050s, and 2080s) climates, resulting in one baseline distribution model and 18 future distributions (= 3 GCMs × 2 emission scenarios × 3 time periods).

### Spatial analysis of baseline and future distributions

We calculated baseline and future habitat area by multiplying the number of raster grid cells considered “suitable” in each predicted distribution by the area of each grid cell (0.01 km^2^). We assessed the potential effects of climate change on predicted distributions by evaluating potential net range change. Range change (RC) was calculated using the formula

RC = 100 × (RG – RL) / BR

where RG = range gain, RL = range loss, and BR = baseline range area. RG and RL were both summed at the level of individual grid cells. We also calculated the number of suitable habitat patches and mean patch size based on the configuration of baseline and future suitable habitat.

## Results

**Fig 3:**
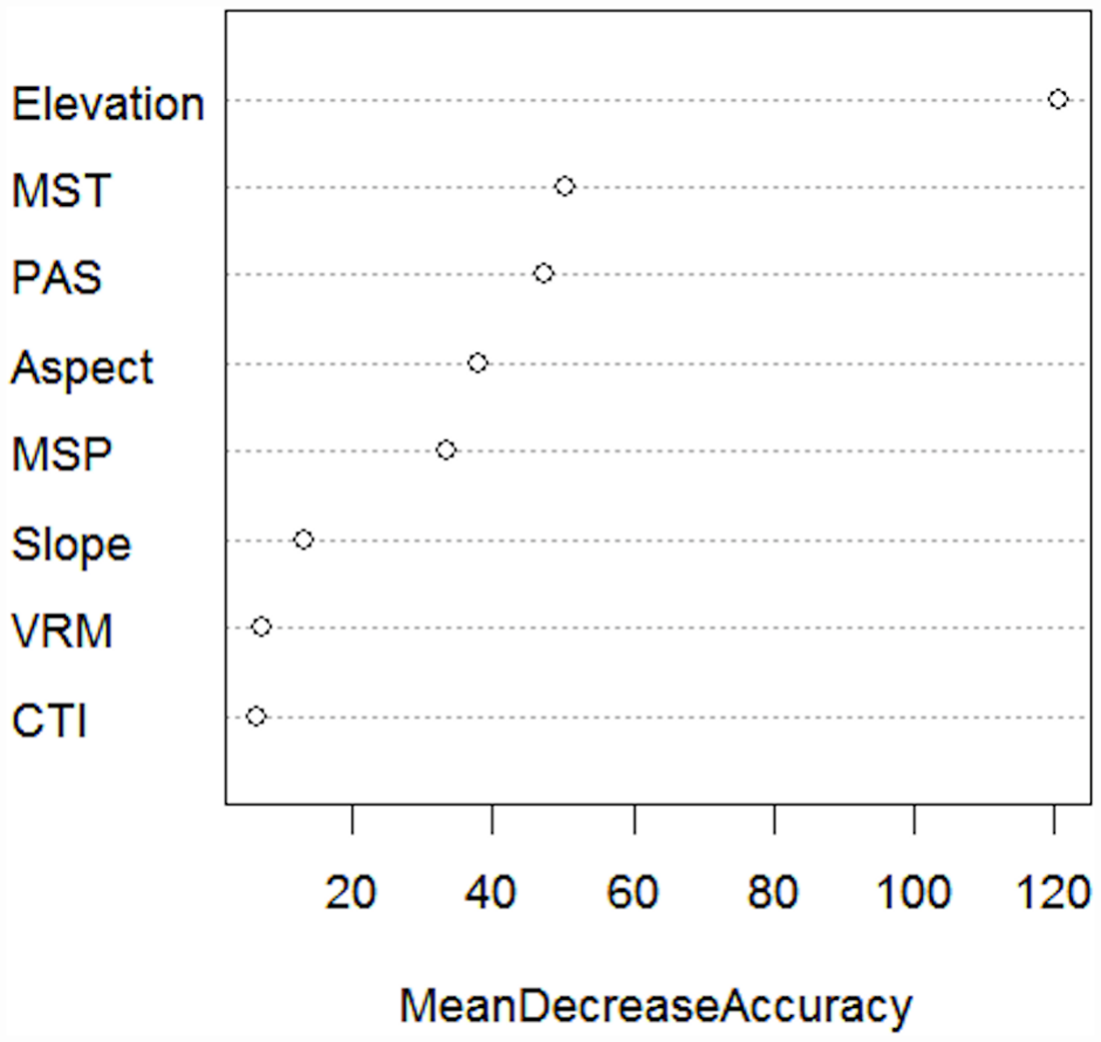
Variable importance plot for predictor variables from the Random Forest model for predicting presence of White-tailed Ptarmigan on Vancouver Island. The mean decrease in accuracy for a variable is the classification accuracy for the out-of-bag data [49]. Variables include elevation, mean summer temperature (MST), precipitation as snow (PAS), aspect, mean summer precipitation (MSP), slope, vector ruggedness measure (VRM), and compound topographic index (CTI). See Table 1 for variable descriptions.

According to the Random Forest model, elevation was the most important variable for predicting White-tailed Ptarmigan habitat (Fig 3, S1 Fig). Mean summer temperature, precipitation as snow, aspect, and mean summer precipitation all were of intermediate importance, while slope, VRM, and CTI were of low importance.

The threshold-independent area under the receiver-operating curve (AUC) measure of model accuracy revealed high predictive accuracy (AUC = 0.94 ± 0.008). The threshold habitat suitability chosen to maximize model sensitivity at 0.90 was 0.36; all pixels with habitat suitability above the threshold were considered “suitable” and those below the threshold were considered “unsuitable.” Specificity (% of pseudo-absences correctly classified) was 0.86, and the RF model performed within the ‘excellent’ range of the kappa index (kappa = 0.73; [52]).

**Fig 4:**
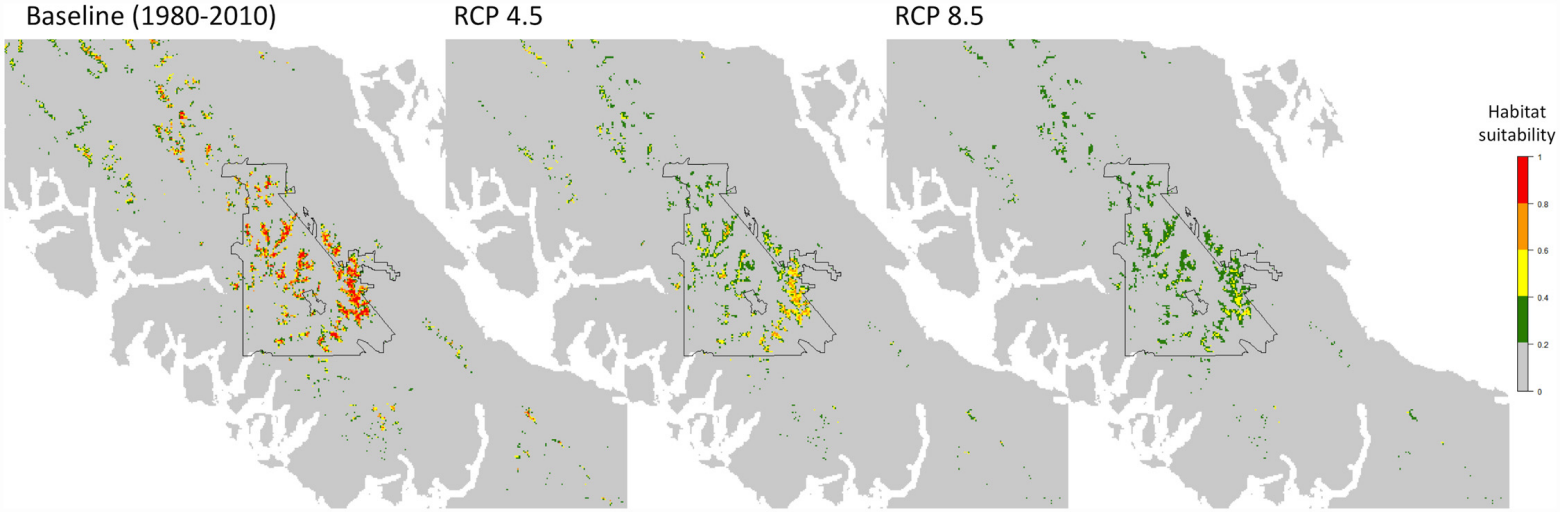
Predicted habitat suitability according to probability of presence (%) using a Random Forest model for Vancouver Island White-tailed Ptarmigan under baseline climate (1980–2010 normals) and future climate (2080s). Future habitat suitability represents averages across 3 general circulation models (CanESM2, CCSM4, and GFDL-CM3) for 2 IPCC greenhouse gas concentration scenarios (RCP 4.5 and RCP 8.5) projected out to the 2080s. Strathcona Provincial Park is outlined in black.

Habitat suitability under baseline climate was greatest at high elevations inside Strathcona Provincial Park in the center of Vancouver Island, with small patches of highly suitable habitat in the northern and southern mountains (Fig 4). Habitat suitability declined across all of Vancouver Island by the 2080s under both RCP scenarios. Most of ptarmigan baseline range is predicted to contain only low suitability habitat by the 2080s under the highest greenhouse gas scenario (RCP 8.5). Habitat suitability is predicted to remain highest within the Forbidden Plateau region in the southeast corner of Strathcona Provincial Park, with a fairly large patch of moderately to highly suitable habitat remaining by 2085 under the lower greenhouse gas scenario but only a few small patches of moderately suitable habitat remaining under the high greenhouse gas scenario.

**Fig 5:**
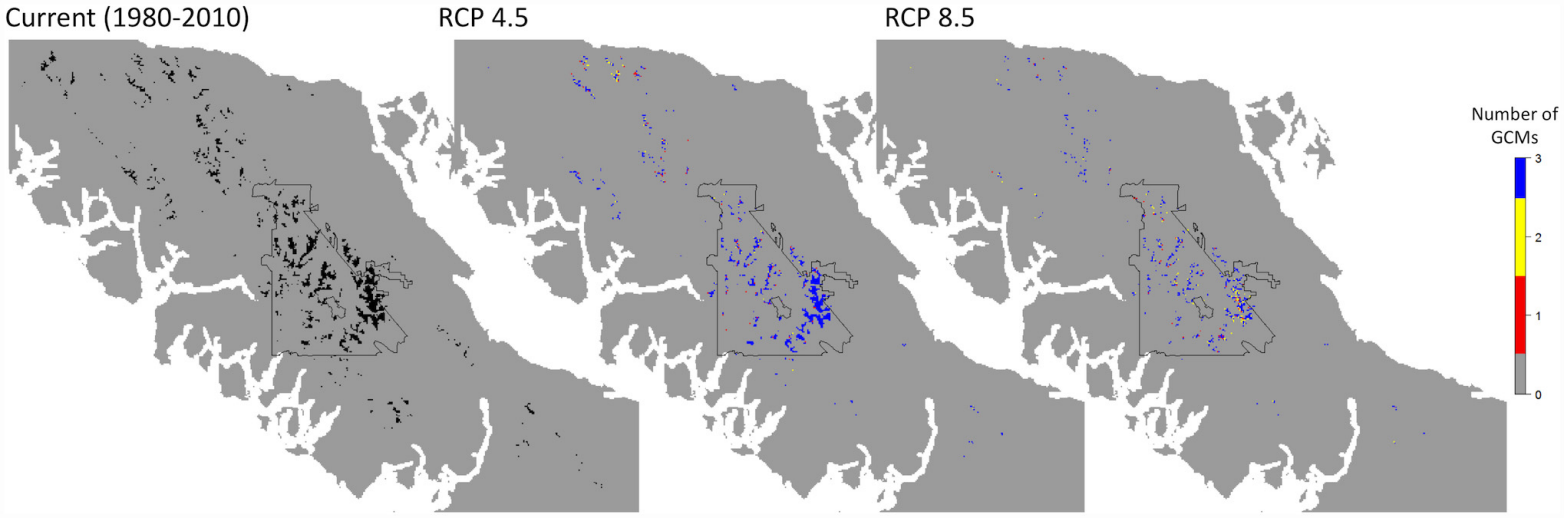
Distribution of “suitable” VIWTP habitat according to a threshold that maximizes sensitivity at 0.9 for Vancouver Island White-tailed Ptarmigan under baseline climate (black), and the number of general circulation models (GCMs) that predict suitable habitat under future climate scenarios by the 2080s under two IPCC greenhouse gas concentration scenarios (RCP 4.5 and RCP 8.5) . Strathcona Provincial Park is outlined in black.

**Fig 6:**
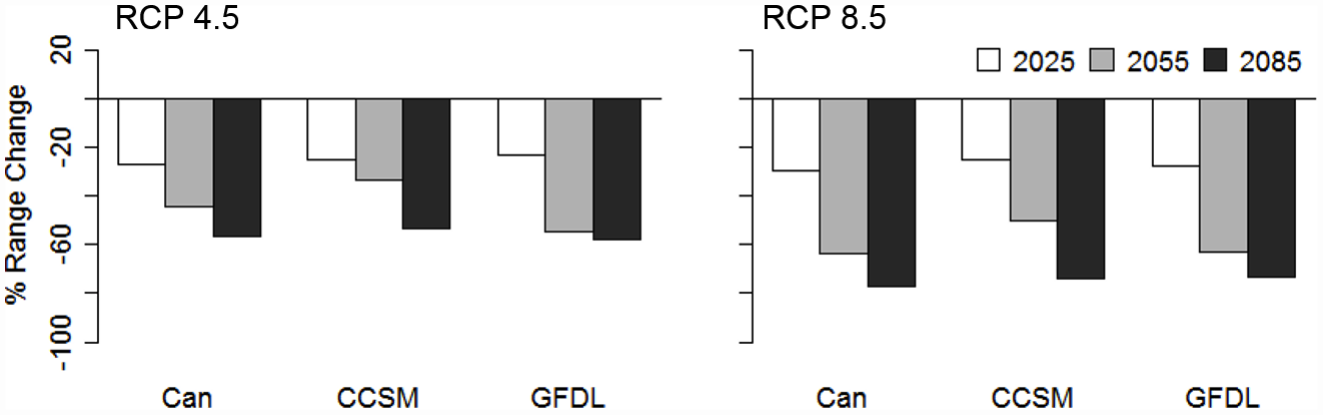
Percentage of range change (100 x [range gain–range loss]/baseline range) by the 2020s (2025), 2050s (2055), and 2080s (2085) according to three general circulation models: CanESM2 (Can), CCSM4 (CCSM), and GFDL-CM3 (GFDL), and two IPCC greenhouse gas concentration scenarios: RCP 4.5 and RCP 8.5.

Baseline suitable habitat according to the threshold encompassed 521 km^2^ of Vancouver Island and dropped to 229 (± 13) km^2^ under the RCP 4.5 scenario and 132 (± 10) km^2^ under the RCP 8.5 scenario by the 2080s (Fig 5). Habitat decline is predicted to occur gradually, with an average range change of -25% (± 2), -44% (± 11), and -56% (± 2) by the 2020s, 2050s, and 2080s, respectively, under the RCP 4.5 scenario and -27% (± 2), -59% (± 8), and -74% (± 2) under the RCP 8.5 scenario (Fig 6). Predictions of range change differed markedly under the two greenhouse gas scenarios; more habitat was predicted to be lost by the 2050s under the RCP 8.5 scenario than by the 2080s under the RCP 4.5 scenario. The three general circulation models (GCMs) showed strong agreement in their predictions of the future distribution of suitable ptarmigan habitat and indicated that remaining suitable habitat will be located within Strathcona Provincial Park, with little to no remaining habitat in the southern ranges and only small, isolated habitat patches in the northern ranges by 2085 (Fig 5).

**Fig 7:**
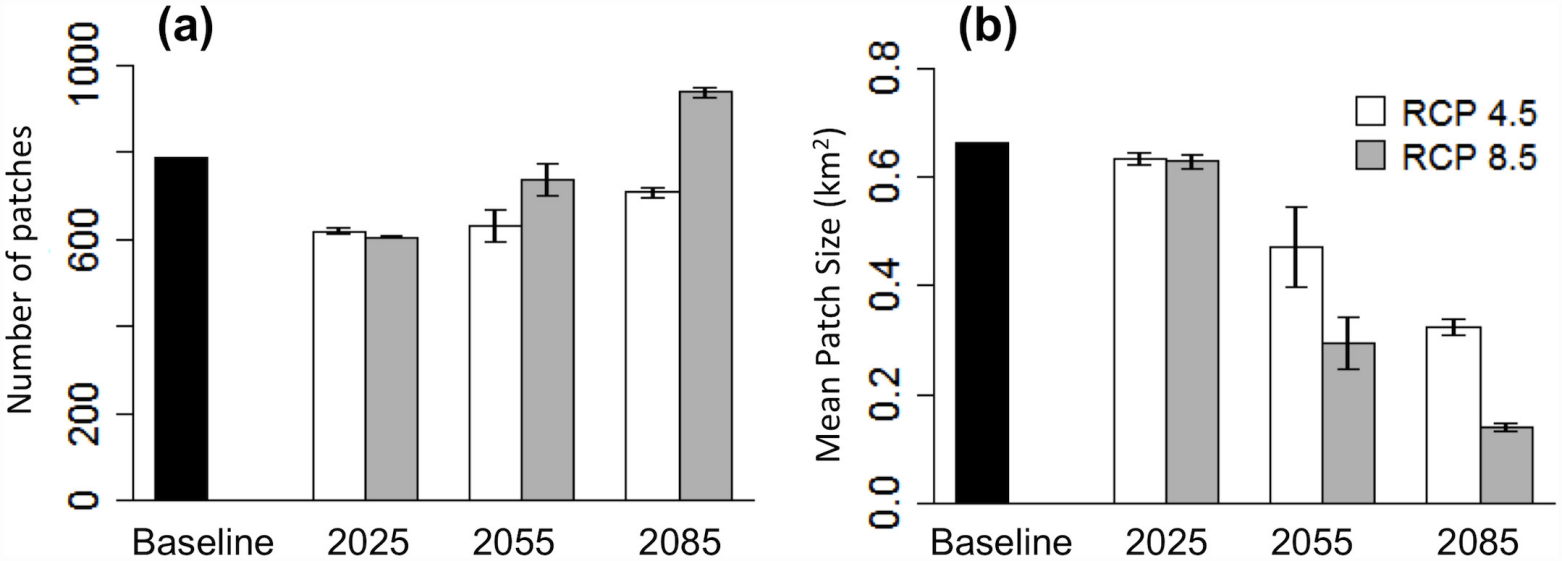
Number (a) and mean size (b) of suitable ptarmigan habitat patches under baseline climate (1980–2010 normals) and by the 2020s (2025), 2050s (2055), and 2080s (2085). Future values represent averages across three general circulation models: CanESM2 (Can), CCSM4 (CCSM), and GFDL-CM3 (GFDL), and are presented for two IPCC greenhouse gas concentration scenarios: RCP 4.5 and RCP 8.5. Error bars represent 1 SE.

**Fig 8:**
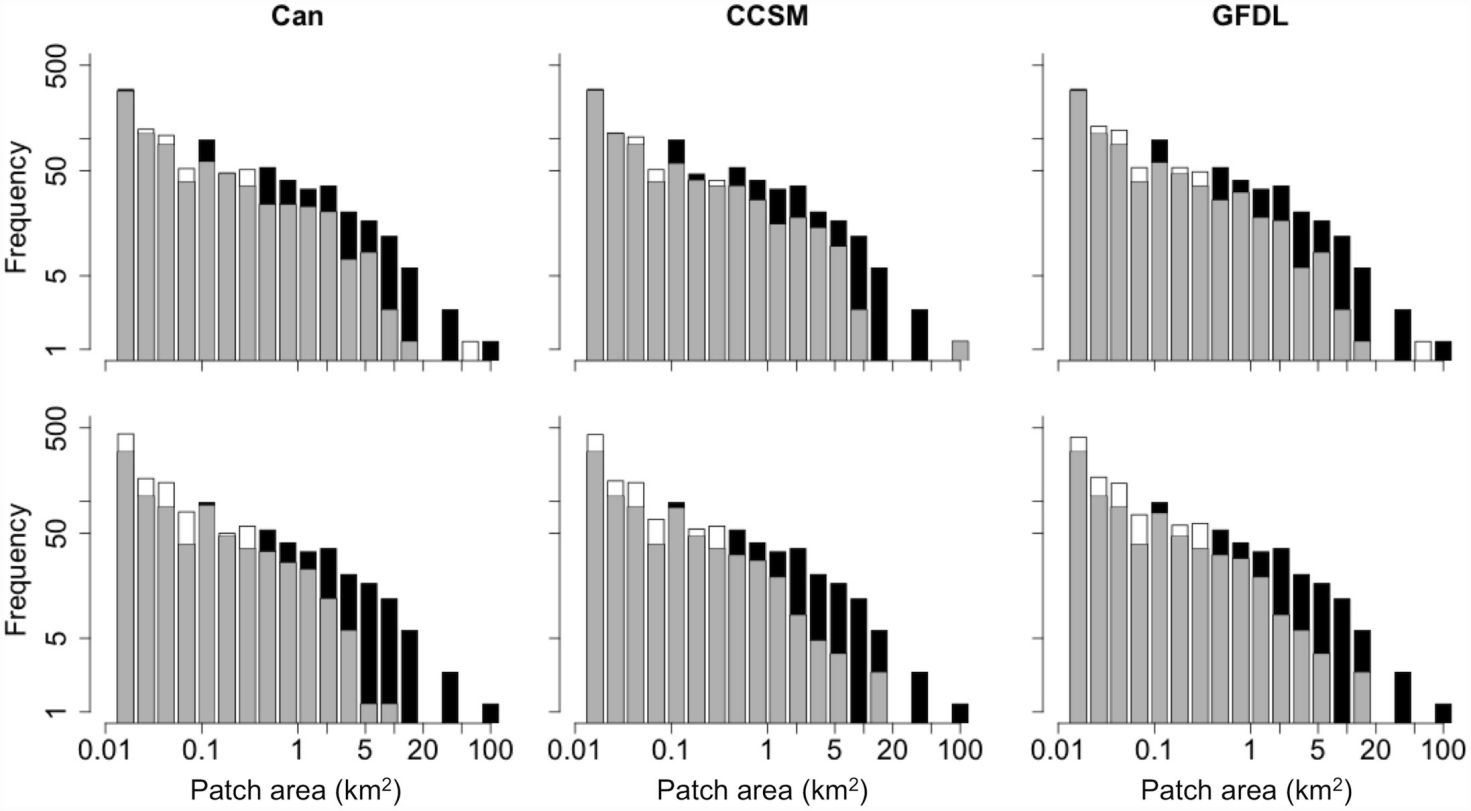
Histograms of the range of suitable ptarmigan habitat patch areas (km^2^). Baseline climate (1980–2010) is shown in black, future climate for the 2080s is shown in white, and their overlap is shown in gray. Future climate is represented by three general circulation models: CanESM2 (Can), CCSM4 (CCSM), and GFDL-CM3 (GFDL), and two emissions scenarios (top row = RCP 4.5 and bottom row = RCP 8.5).

**Fig 9:**
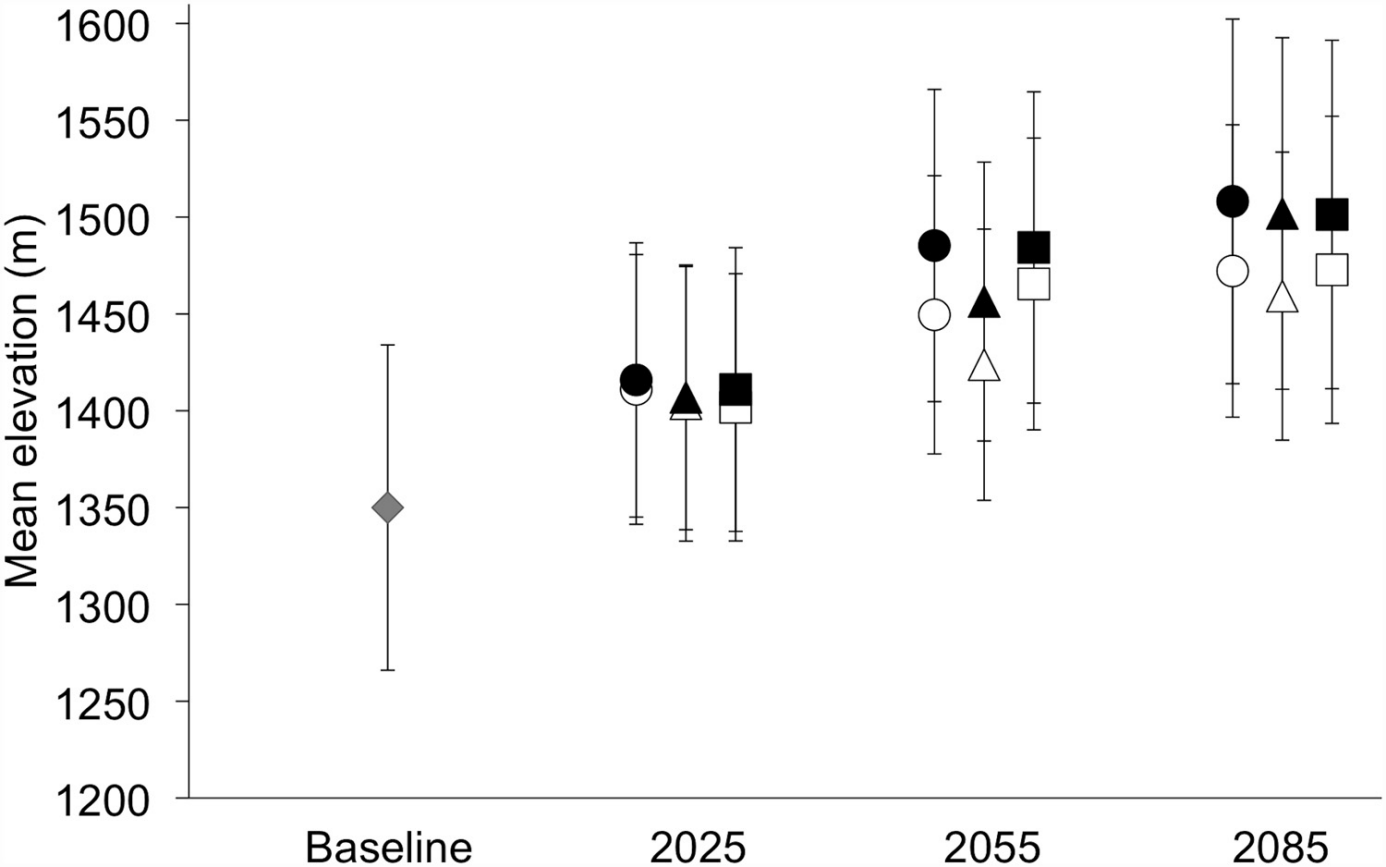
Mean elevation (m) of suitable ptarmigan habitat patches under baseline climate (1980–2010 normals) and by the 2020s (2025), 2050s (2055), and 2080s (2085) for three general circulation models (circles = CanESM2, squares = CCSM4, triangles = GFDL-CM3) and two IPCC greenhouse gas concentration scenarios (open symbols = RCP 4.5, closed symbols = RCP 8.5). Error bars represent 1 SD.

Number of patches is predicted to decline by the 2020s followed by an increase by the 2080s under both greenhouse gas scenarios (Fig 7). This increase in number of patches corresponds with a 52% reduction in mean patch size from 6.6 km^2^ to 3.2 (± 0.03) km^2^ under the RCP 4.5 scenario and a 79% reduction in mean patch size to 1.4 (± 0.01) km^2^ under the RCP 8.5 scenario by the 2080s. Currently large patches such as those in the Forbidden Plateau, southeast Strathcona Park (Fig 5), are predicted to become fragmented into several smaller patches. In particular, the three currently largest patches of suitable habitat are predicted to become fragmented into several smaller patches by the 2080s (Fig 8). The largest patch by the 2080s accounts for 25–29% of the predicted remaining habitat area under the RCP 4.5 scenario and 5–9% of predicted remaining habitat area under the RCP 8.5 scenario. The Can GCM and RCP 8.5 greenhouse gas scenario depict the most dire predictions of habitat change for White-tailed Ptarmigan, with total loss of all habitat patches larger than 6 km^2^ and 97% of all remaining habitat patches < 1 km^2^. Average elevation of suitable habitat patches is predicted to increase from 1,350 m (± 84) under the baseline climate to 1,459–1,472 m under the RCP 4.5 scenario and 1,501–1,508 m under the RCP 8.5 scenario by the 2080s (Fig 9).

## Discussion

Our approach predicts that Vancouver Island White-tailed Ptarmigan may lose a substantial portion of their habitat as early as the 2020s, as patches become smaller and increasingly fragmented. The GCMs differed very little in their estimates of habitat loss and configuration, whereas greenhouse gas concentration scenarios depicted very different futures for VIWTP habitat. By the 2080s, we predict VIWTP will lose at least 53%, or as high as 77%, of their habitat under the low and high greenhouse gas scenarios, respectively. Our predictions are consistent with predictions of significant range contractions for other high elevation specialist species as a consequence of global climate change [12,14,53,54].

### Warmer summers and reduced snowfall drive VIWTP range change

After elevation, mean summer temperature and precipitation as snow were the second and third most important variables for predicting VIWTP distributions. Changes in these climate variables will likely drive habitat loss. Increased annual mean summer temperature will likely correspond with increased frequency of extreme heat days, which may stress birds and lead to reduced fecundity or survival [30,31]. Warmer summers and reduced snowfall will also lead to the loss of permanent snow fields, which are important for ptarmigan thermoregulation and for providing sufficient moisture for ptarmigan forage [31,32,55]. Additionally, ptarmigan moult is primarily influenced by photoperiod and not climate [56]. As the number of snow-free days during the year increase, birds with white plumage against a dark background may be at increased risk of predation, especially independent but naive juveniles. Birds may adapt to changing conditions through evolutionary changes in their molt response to photoperiod and increased ability to tolerate heat, but such selective responses are likely to be slow [57].

### Climate change greatly impacts spatial configuration of VIWTP habitat

While many studies project future range shifts for wildlife, the spatial configuration and connectivity of future habitats has been less well addressed. Our work predicts that VIWTP habitat will become increasingly fragmented and confined to mountaintops. Climate change will likely cause fragmentation of VIWTP habitat by facilitating infilling of trees and shrubs into previously open alpine tundra [8,9,58], a process already documented at high elevations on Vancouver Island [25,26]. The effects of habitat fragmentation on VIWTP persistence will depend on their home range size and dispersal ability. Substantial movement of VIWTP genes occurs across the landscape and high allelic richness is found even among the most isolated populations [59]. Female White-tailed Ptarmigan in Colorado can move long distances to other breeding areas without incurring reproductive or survival costs during the breeding season [34]. These studies suggest that ptarmigan may be able to persist on Vancouver Island despite increasing habitat fragmentation due to their apparent ability to move between patches, at least for the initial periods of change. Still, our results predict the disappearance of nearly all of the ptarmigan habitat south of Strathcona Provincial Park by the 2080s. Large areas of unsuitable low elevation habitat act as a barrier to VIWTP gene flow, as the southern populations appeared to be smaller and more temporally variable [59]. Thus, these southern populations may well be extirpated by climate-induced habitat loss within 70 years. The ability of ptarmigan to disperse between smaller and more spatially fragmented habitat patches will be an important predictor of VIWTP long-term viability, and future research should examine VIWTP dispersal distance as a function of future climates in order to inform management and conservation of the subspecies.

Under all climate change scenarios, our results predict substantial decline in the average suitable patch size for VIWTP by the 2080s, including loss of most large patches and an increasing number of very small patches. Although average patch size is predicted to decline gradually from the 2020s–2080s, total number of patches is predicted to decline by the 2020s but then increase in the 2050s and 2080s. The predicted reduction in number of patches by the 2020s primarily reflects the disappearance of most very small patches of currently suitable habitat. By the 2080s, however, most current larger patches are predicted to become fragmented into several smaller patches, increasing the total number of patches but lowering average patch size. As shown by [33], there is likely a demographic cost for VIWTP persisting in smaller patches. Alpine habitat already exists as small, fragmented “sky islands” on Vancouver Island compared to interior alpine, which is more continuous. Further fragmentation and reduction in patch size of VIWTP habitat will likely be detrimental to ptarmigan breeding success [33] and may also hinder dispersal and increase the ecological cost of movement, as birds will increasingly be forced to cross unsuitable habitat in order to reach suitable breeding patches. Finally, the average elevation of suitable habitat patches is expected to increase by the 2080s, reflecting a loss of patches at lower elevations. Thus ptarmigan are in danger of being “squeezed off the mountain”, as their remaining suitable habitat will be increasingly confined to mountaintops.

### Implications for VIWTP population persistence and the importance of refugia

The configuration of future habitat is important when modeling shifts in climate envelopes in part because of the importance of micro- and macrorefugia in allowing the persistence of populations. VIWTP may show resilience to climate change-induced habitat loss through the use of microrefugia. Microrefugia differ from macrorefugia in that they are small areas with unusual microclimates that allow species to persist in areas that might be deemed unsuitable according to average background climates [60,61]. Coarse scale species distribution models tend to overestimate extinction risk because they use climate grids that are too coarse to predict locations of fine-scale topographic relief that may provide microrefugia [62,63]. Although we used a smaller climate grid (100 m) than many species distribution modeling studies, we acknowledge that we can still only identify climate macrorefugia. Randin *et al*. [64] found that local-scale models (25 m grid cells) predicted some persistence of suitable habitats at high elevations for European plant species that were predicted to lose all of their suitable habitat according to large-scale models (10′ grid cells). Similarly, ptarmigan may be able to utilize microclimates created by fine-scale landscape features (e.g., boulders, ravines, and water bodies), vegetation type, or small variations in slope or aspect not accounted for in our models to persist in habitat that is predicted to be lost (see [65,66]). In contrast, Trivedi *et al*. [67] found that coarse scale models overestimated species thermal tolerances and underestimated the potential impact of climate change on high elevation species compared to local-scale models, suggesting that microclimatic refugia may not allow for persistence as speculated.

Even if ptarmigan can utilize microrefugia, it is unlikely that populations will be able to persist in their current numbers. Extinction probability increases with reduced range size and connectivity [68], and climate change will increase the variability of extreme temperatures and weather events in high elevation habitats [69]. Ptarmigan must be able to live and reproduce through these extreme episodes if they are to persist. Alpine birds tend to exhibit slower life histories than low elevation populations of the same or similar species, where high elevation individuals have the lowest breeding success but higher survival [70,71]. More frequent episodes of extreme conditions may alter the fitness consequences of alternative life-history strategies and exceed the coping abilities of alpine-breeding birds.

The persistence of populations in the central and northern regions of Vancouver Island will depend partially on the ability of birds to utilize smaller areas at higher densities. Alpine ibex (*Capra ibex*) have shown intensified density dependence under unfavourable climatic conditions [72], which also may be the case for ptarmigan especially if the quality of “suitable” habitat declines. For instance, ptarmigan may encounter more competition from lower elevation competitors such as Sooty Grouse (*Dendragapus fuliginosus*) and increased predation pressure from generalist predators such as ravens, particularly around nest sites [73]. This potential replacement of an alpine specialist with generalist competitors and increased predators corresponds to recent findings in Europe, where local species assemblages are increasingly composed of native habitat generalists [74].

### Strengths and limitations of climate envelope models

The choice of a threshold for determining habitat suitability with species distribution models can be subjective, especially when reliable absence data are not available [39,75]. We used a threshold that maximized sensitivity (set at 0.90) to correctly predict the largest number of ptarmigan presence locations while attempting to avoid overpredicting habitat suitability across the landscape by setting sensitivity too high. However, as in most studies of this kind, we cannot be sure that areas without ptarmigan sightings represent unsuitable summer habitat. A series of initial trials revealed that our choice of threshold and resulting estimates of habitat loss were conservative; other methodological choices for setting a threshold, such as maximizing model accuracy according to kappa or setting a lower sensitivity, resulted in smaller estimates of suitable habitat area and greater predictions for climate-induced habitat loss. By utilizing a threshold that slightly over-predicts baseline suitable habitat for VIWTP, we minimized the chances of “missing” areas of suitable habitat and overestimating habitat loss as often occurs in climate envelope studies [76].

Although their shortcomings have been described elsewhere [77,78], predictions from climate envelope models have corresponded well with observed population trends for breeding birds [3,79,80] and range shifts for montane birds [81]. Climate envelope models can also be useful for identifying conservation opportunities within climatic macro-refugia or newly available habitat [76]. Our predictions for VIWTP appear realistic based on predictions for loss of alpine tundra in British Columbia [37], and allow for informed decisions for prioritizing management of areas where ptarmigan are likely to persist.

## Conclusions

Changes in the configuration of alpine habitat, including fragmentation and increasing confinement to high elevation mountaintops, will likely play a large role in driving the responses of alpine wildlife to climate change. VIWTP, along with other alpine specialists, are in danger of being “squeezed off the mountain” as alpine habitat declines in total area and mean patch size. Our results also emphasize that regardless of changes in habitat size and connectivity and the threshold chosen to define “suitable” ptarmigan habitat, habitat quality will be reduced from the birds’ point of view. This reduction in quality may manifest in several ways including physiological intolerance to warming, reduced survival or brood failure due to increased frequency of extreme weather events, lower food availability, and/or increased competition or predation. Little is known about the potential fate of North America’s coastal alpine ecosystems under climate change or the ability of alpine animals to cope with deteriorating habitat quality. Because our study has implications for other high elevation specialist species with similarly restricted ranges, White-tailed Ptarmigan can be considered an indicator species for coastal alpine ecosystems in the face of climate change.

## Supporting Information

S1 Fig Mean decrease in Gini Index. The mean decrease in the Gini index as a measure of variable importance for predictor variables from the Random Forest model for predicting presence of White-tailed Ptarmigan on Vancouver Island. Variables include elevation, mean summer temperature (MST), precipitation as snow (PAS), aspect, mean summer precipitation (MSP), slope, vector ruggedness measure (VRM), and compound topographic index (CTI). See Table 1 for variable descriptions.
(TIFF)
