# Correction: Effects of Climate Change on Habitat Availability and Configuration for an Endemic Coastal Alpine Bird

**DOI:** 10.1371/journal.pone.0146838

**Published:** 2016-01-05

**Authors:** Michelle M. Jackson, Sarah E. Gergel, Kathy Martin

There is an error in the second sentence of the fifth paragraph of the Results section. The correct sentence is: This increase in number of patches by the 2080s corresponds with a 52% reduction in mean patch size from 0.66 km^2^ to 0.32 (± 0.03) km^2^ under the RCP 4.5 scenario and a 79% reduction in mean patch size to 0.14 (± 0.01) km^2^ under the RCP 8.5 scenario.
