# Supplementary figures and images for: Effects of Climate Change on Habitat Availability and Configuration for an Endemic Coastal Alpine Bird
Source: PLoS One. 2015 Nov 3;10(11):e0142110. doi: 10.1371/journal.pone.0142110 (PMC4631505; doi:10.1371/journal.pone.0142110)

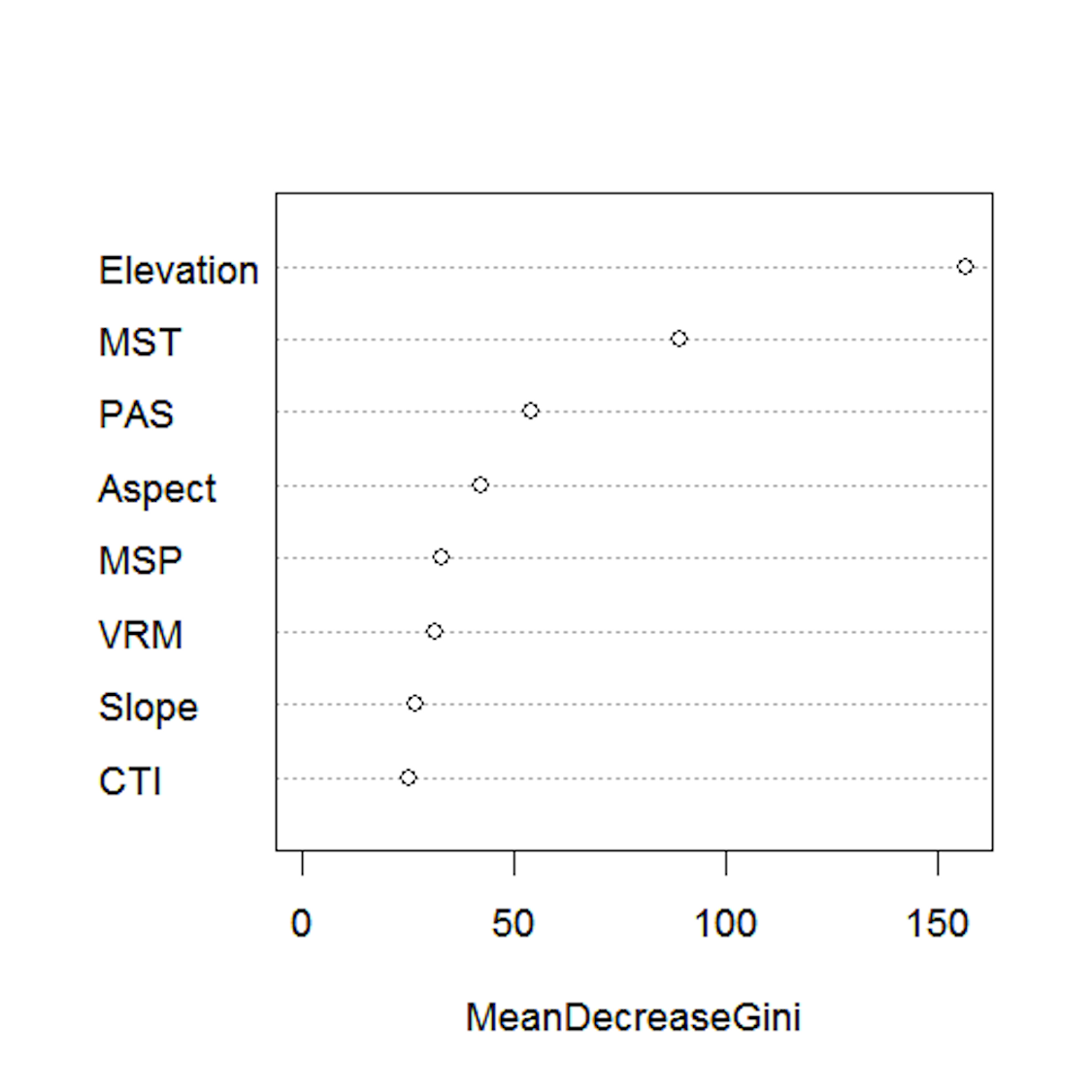

Supplement: S1 Fig — The mean decrease in the Gini index as a measure of variable importance for predictor variables from the Random Forest model for predicting presence of White-tailed Ptarmigan on Vancouver Island. Variables include elevation, mean summer temperature (MST), precipitation as snow (PAS), aspect, mean summer precipitation (MSP), slope, vector ruggedness measure (VRM), and compound topographic index (CTI). See Table 1 for variable descriptions. (TIFF) [file pone.0142110.s001.tiff]
